# Embedding research to improve program implementation in Latin America and the Caribbean

**DOI:** 10.26633/RPSP.2017.75

**Published:** 2017-04-28

**Authors:** Nhan Tran, Etienne V Langlois, Ludovic Reveiz, Ilona Varallyay, Vanessa Elias, Arielle Mancuso, Francisco Becerra-Posada, Abdul Ghaffar

**Affiliations:** 1 Alliance for Health Policy and Systems Research World Health Organization Geneva Switzerland Alliance for Health Policy and Systems Research, World Health Organization, Geneva, Switzerland.; 2 Office of Knowledge Management, Bioethics and Research Pan American Health Organization Washington, D.C. United States of America Office of Knowledge Management, Bioethics and Research, Pan American Health Organization, Washington, D.C., United States of America.; 3 Johns Hopkins University Bloomberg School of Public Health Baltimore Maryland United States of America Johns Hopkins University Bloomberg School of Public Health, Baltimore, Maryland, United States of America.

**Keywords:** Evidence-based practice, health plan implementation, program evaluation, program evaluation, West Indies, Práctica clínica basada en la evidencia, implementación de plan de salud, evaluación de programas y proyectos de salud, América Latina, Indias Occidentales, Prática clínica baseada em evidências, implementação de plano de saúde, avaliação de programas e projetos de saúde, América Latina, Índias Ocidentais

## Abstract

In the last 10 years, implementation research has come to play a critical role in improving the implementation of already-proven health interventions by promoting the systematic uptake of research findings and other evidence-based strategies into routine practice. The Alliance for Health Policy and Systems Research and the Pan American Health Organization implemented a program of embedded implementation research to support health programs in Latin America and the Caribbean (LAC) in 2014–2015. A total of 234 applications were received from 28 countries in the Americas. The Improving Program Implementation through Embedded Research (iPIER) scheme supported 12 implementation research projects led by health program implementers from nine LAC countries: Argentina, Bolivia, Brazil, Chile, Colombia, Mexico, Panama, Peru, and Saint Lucia. Through this experience, we learned that the “insider” perspective, which implementers bring to the research proposal, is particularly important in identifying research questions that focus on the systems failures that often manifest in barriers to implementation. This paper documents the experience of and highlights key conclusions about the conduct of embedded implementation research. The iPIER experience has shown great promise for embedded research models that place implementers at the helm of implementation research initiatives.

## RESUMEN

En los últimos 10 años, las investigaciones sobre la ejecución de programas han desempeñado una función crucial en lo que respecta a la mejora de la ejecución de intervenciones de salud de eficacia comprobada, porque han promovido la incorporación sistemática de los resultados de investigaciones y otras estrategias basadas en datos probatorios en la práctica cotidiana. Entre el 2014 y el 2015, la Alianza para la Investigación en Políticas y Sistemas de Salud, y la Organización Panamericana de la Salud pusieron en marcha un proyecto que propone incorporar las investigaciones sobre la ejecución para apoyar a los programas de salud en América Latina y el Caribe. En total, se recibieron 234 solicitudes de 28 países de las Américas. La iniciativa “Mejora de la Ejecución de los Programas mediante la Incorporación de la Investigación (iPIER)” respaldó la realización de 12 proyectos de investigación dirigidos por ejecutores de programas de salud de nueve países de América Latina y el Caribe: Argentina, Bolivia, Brasil, Chile, Colombia, México, Panamá, Perú y Santa Lucía. Gracias a esta experiencia, somos conscientes de que la perspectiva “desde dentro”, con la que contribuyen los ejecutores a la propuesta de investigación, es particularmente importante para definir las preguntas de investigación que apuntan a las deficiencias de los sistemas a menudo manifestadas a través de obstáculos que dificultan la ejecución. Este artículo documenta la experiencia derivada de las investigaciones incorporadas en la ejecución de los programas y destaca las conclusiones más importantes. La experiencia iPIER resulta muy prometedora para los modelos de ejecución que proponen incorporar las investigaciones y que colocan a los ejecutores al mando de estas iniciativas.

## RESUMO

Na última década, a pesquisa em implementação tem tido papel fundamental em melhorar a implementação de intervenções de saúde comprovadas com o incentivo à absorção metódica dos resultados de pesquisas e outras constatações na prática diária. A Aliança para Pesquisa em Políticas e Sistemas de Saúde e a Organização Pan-Americana da Saúde criaram um programa de pesquisa em implementação integrada para dar respaldo aos programas de saúde na América Latina e no Caribe (ALC) em 2014–2015. Foram recebidas ao todo 234 propostas provenientes de 28 países nas Américas. A iniciativa Melhorar a Implementação de Programas com Pesquisa Integrada (iPIER) subsidiou 12 projetos de pesquisa em implementação conduzidos pelos responsáveis pela execução dos programas públicos de saúde de nove países da ALC: Argentina, Bolívia, Brasil, Chile, Colômbia, México, Panamá, Peru e Santa Lúcia. Esta experiência ensinou que a perspectiva dos “insiders” (pessoal interno), que os responsáveis pela execução dos programas imprimem à proposta da pesquisa, é essencial para identificar a problemática a ser pesquisada com foco nas falhas dos sistemas, que em geral se manifestam como obstáculos à implementação. Neste artigo se destacam a experiência e as principais conclusões sobre a realização de pesquisa em implementação integrada. A experiência iPIER demonstrou serem bastante promissores os modelos de pesquisa integrada quando os responsáveis pela execução dos programas assumem as rédeas das iniciativas de pesquisa em implementação.

In the last 10 years, there has been an increasing emphasis on implementation of known, effective interventions, as it is clear that greater health gains could be achieved by simply improving the implementation of already-proven interventions (1). A key to this is implementation research (IR). Through its focus on promoting the systematic uptake of research findings and other evidence-based strategies into routine practice (2), IR can help to strengthen implementation and inform the scale-up of such interventions, ultimately contributing to global health gains (2, 3).

Since 2010, the Alliance for Health Policy and Systems Research (AHPSR), which is an international partnership housed by the World Health Organization (WHO), in coordination with a number of partners, has supported IR by establishing as a grant requirement that implementers be collaborators on research projects. Though IR had been conducted well by researchers, in some instances it was challenging to ensure that the researchers’ proposed research questions were aligned with the implementers’ program needs and the specific problems experienced within the health system.

The AHPSR has learned that implementers have a more central role to play, particularly in the identification of research questions, given that the implementers have deeper insights into the systems failures that often manifest in barriers to implementation (4). Critical to achieving this level of understanding is the meaningful engagement and participation of those actors within the system who can bring their “insider” perspective to bear on the research being conducted. Recognizing the potential benefits of engaging implementers in IR, the AHPSR decided to explore an approach whereby health sector implementers served as principal investigators. This paper highlights the rationale behind this approach and outlines the many benefits it brings to health program improvement efforts.

## EMBEDDING IMPLEMENTATION RESEARCH TO IMPROVE ITS EFFECTIVENESS

Based on these lessons, the AHPSR has developed an innovative approach of embedded implementation research to support health program improvement in low- and middle-income countries (LMICs). Embedded research aims to shine a light on implementation barriers and associated health systems failures, by engaging actors working within health care systems to conduct rigorous scientific inquiry. The defining feature of the embedded model is that the research process is led by implementers who are able to ensure that the research focuses on real-world implementation priorities. Those implementers possess tacit knowledge of the system and the context in which the program is implemented. They have the authority to make decisions regarding implementation and are also well positioned within the system to foster the political support needed to implement these changes. Furthermore, their involvement allows for the possibility of evaluating impact as part of their program through an established monitoring and evaluation process. These implementers are the stakeholders who are best positioned to ensure that the IR is anchored in health program practice and context and that the research findings are used and integrated in real time in order to support the implementation and scaleup of health programs.

Research embedded in the real world integrates scientific inquiry into the implementation problem-solving process, including programmatic improvements,in an iterative and continuous manner. Embedded research thus incorporates the systematic assessment of implementation barriers, facilitators, and strategies as an integral part of the program process. Embedded research also promotes the scale-up of interventions and their integration into health systems at both the national and subnational level. In addition, the embedded research model emphasizes strong collaboration and continuous exchanges between the implementers leading the study and the researchers specialized in health policy and systems research and implementation research. As coproducers of research, implementers and researchers each have a specific role in ensuring the relevance and applicability of the research conducted, as well as in making certain that the findings generated are used to inform program/policy decision-making processes.

## IMPROVING PROGRAM IMPLEMENTATION THROUGH EMBEDDED RESEARCH (iPIER)

The AHPSR and the Pan American Health Organization (PAHO) implemented a program of embedded implementation research to support health programs in Latin America and the Caribbean (LAC) in 2014–2015. The initiative, entitled (in the Americas) Improving Program Implementation through Embedded Research (iPIER), aimed to support the development of and demand for research that is action oriented and focused on systems-level problems. The iPIER initiative placed implementers at the center of a research inquiry, aiming to understand failures in the health systems that create barriers to implementation, as well as aiming to identify practical, feasible solutions to these barriers. Implementers were engaged as key actors in the research teams in order to conduct and facilitate the use of research on program implementation to effect iterative program improvements.

The iPIER initiative also included a capacity-strengthening component intended to develop the implementers’ abilities to identify implementation barriers, define implementation research questions, conduct implementation research, and integrate implementation research findings into program implementation and health systems strengthening.

In addition to the collaboration with academic institutions, implementers and their research partners received ongoing technical and scientific support from AHPSR, PAHO, and a regional technical assistance center in Argentina, the Institute for Clinical Effectiveness and Health Policy (Instituto de Efectividad Clínica y Sanitaria, IECS).

In 2014, AHPSR and PAHO issued an open call for research proposals restricted to LMIC-based institutions, with the requirement that each proposal’s principal investigator be a health systems implementer. For the purpose of the call, “implementers” were broadly defined: “[They are] key stakeholders involved in policy generation and/or program management, i.e., implementers at the forefront of specific implementation problems as well as the contexts in which they occur. They can include implementers responsible for designing policies and managing programs whose decisions shape implementation and scale-up processes, as well as practitioners who ultimately implement these decisions. Program managers, district health officers, and front-line health workers are typical examples of such individuals.”

In total, 234 applications were received, from 28 countries in the Americas. After external review and a thorough adjudication process, the iPIER scheme supported seven initial implementation research projects led by health program implementers from six LAC countries: Argentina, Brazil, Chile (two projects), Colombia, Mexico, and Peru. In 2015, PAHO made five additional grants, to Argentina, Bolivia, Chile, Panama, and Saint Lucia. Each team received a maximum subsidy of US$ 30 000 to finance implementation research activities over 9 to 12 months.

The 2014 cohort of country teams was invited for a one-week protocol development workshop held in Washington, D.C., in December 2014 to develop robust and scientifically sound implementation research proposals. At that event, each implementer leading the study was accompanied by a researcher from the institution identified as the co-applicant on the iPIER grant. A wide range of implementers working at different levels of the health system participated in this workshop, including managers of specific programs such as TB and reproductive health; health policy planners; high-level policymakers; and even a provincial minister of health. The workshop included training sessions on key implementation research methods, use of IR findings to improve health program implementation and performance of health systems, research project management, and compliance with the WHO/PAHO ethical review process.

With direct support from AHPSR, PAHO, and IECS, the implementerresearcher dyads worked on developing a first draft of the embedded research protocol. The protocol identified systems-level failures as well as the knowledge needed to understand and respond to those systems failures. Such details are not always explicit in other research proposals; this attribute was one of the defining features of this approach.

**FIGURE 1.**
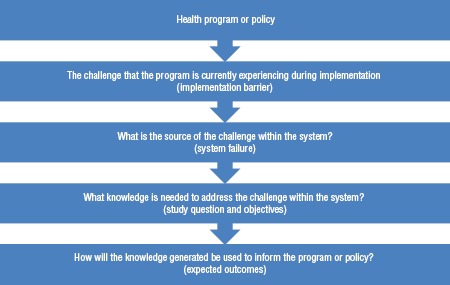
Flow chart that grant-winning country teams in the Americas developed to guide their Improving Program Implementation through Embedded Research (iPIER) projects

The country teams also developed a flow chart (Figure 1), which aimed at thinking upfront about the impact of the implementation research conducted, as well as pathways through which the IR findings could inform iterative improvements in health program implementation. Many grantees were not accustomed to these aspects of the research process. Therefore, the participants often struggled to fine-tune their protocols to focus on implementation issues in terms of systems failures. In addition, homing in on the last two steps of this flow chart (study questions and expected outcomes) also proved challenging, since this required a different way of thinking about research.

Throughout this first round of grants, it became evident that central to this exercise is a solid understanding of the systems-level failures driving the implementation barriers that are observed. These barriers are often only symptoms of deeper problems in the system. Initially, researchers often tended to focus on the immediate barriers. By engaging implementers in this process, problemsolving discussions were able to go beyond just proximal barriers and delve deeper into the systems failures at the root of these barriers. Without this understanding of the systems failures underlying implementation challenges, it is difficult to devise adequate solutions to these challenges. Furthermore, a critical finding of these first experiences with embedded research was that without the active and vested participation of implementers, productive discussions about these system failures were lacking. This insight reinforced the importance of the role of implementers throughout the IR process, beginning at the earliest stages of research, with the definition of the question and the research design.

The additional five projects, selected from the balance of the original 234 proposals, were funded by PAHO in 2015, following a similar methodological pathway as their predecessors. However, there was one key difference: the oneweek protocol development workshop was carried out in the host country of each of the grant recipients. Staff from the IECS technical assistance center and from PAHO traveled to each of the country sites to discuss barriers and facilitators to executing programs, as well as to develop the implementation research protocols. As with the 2014 meeting in Washington, D.C., in these 2015 in-country sessions, both the implementer acting as the principal investigator and a researcher participated in the workshops. The advantage of having each protocol development workshop in the respective host country was that it also permitted relevant stakeholders invested in the topic to participate for part or all of the meeting, which influenced the process of obtaining buy-in from key actors in the broader decision-making realm.

Following the completion of the study protocol, grantees sought approval from a local ethical review board and the PAHO Ethical Review Board. Data collection activities were supported by IECS and PAHO.

The data collected were then reviewed and used during the second major iPIER capacity-strengthening activity: a data analysis workshop. The seven initial teams gathered in the city of Rosario, Argentina, in June 2015 to work on the IR data and to reflect and exchange ideas on the use of findings to bring about improvements in program implementation processes. The second round of grantees carried out a similar data analysis workshop in Washington, D.C., in November 2015.

Both the Rosario and Washington, D.C., data analysis workshops provided the opportunity for the country teams to learn about specific methods of research data analysis and the use of evidence for decision-making in the context of health programs. Presentations on implementation research focused on how to use research results to improve health programs and on the distinct methods of investigation in the context of health programs.

An important feature of the protocol development and data analysis workshops was the exchange of ideas between researchers and implementers. Both groups brought perspectives that enabled a more comprehensive understanding of the systems failures, resulting in the formulation of more targeted implementation research questions. The November 2015 Washington, D.C., data analysis meeting for the second round of grants, also included presentations by IECS on qualitative study design and the analysis of qualitative data. At the data analysis workshops, grantees presented the preliminary results of their IR findings to the group, allowing the country teams to identify shared experiences and learn from the development of results from each proposal.

In addition, the capacity-building sessions during the protocol development and data analysis workshops provided an opportunity to discuss the relevance of complexity science and systems thinking in addressing the health systems failures identified in the studies (3). The protocol development and data analysis workshops provided a space for all stakeholders involved to devise a strategy to integrate IR findings into complex policy and health systems decision-making processes, with guidance and support from AHPSR, PAHO, and IECS.

Drawing from our experience to date, the embedded approach to implementation research will continue to be the cornerstone of implementation research activities funded by AHPSR. In 2016– 2017, AHPSR and the PAHO will implement a third round of iPIER grants in LAC, using this same embedded research model. Following a call for proposals in June 2016, eight new grants were awarded, in eight countries: Argentina, Bolivia, Brazil, Chile, Colombia, the Dominican Republic, Mexico, and Peru.

## KEY LESSONS ON THE EMBEDDED RESEARCH MODEL

Our experience with the iPIER approach in Latin America and the Caribbean yields a great deal of insight on embedded research more broadly. While the concept of “embedded” research is still evolving, a few criteria emerge as fundamental to this approach. First, the role of implementers is paramount for reaping the full benefits of embedded implementation research. In order to produce relevant, action-oriented implementation research, there is a need to understand the context and system in which programs are implemented. This requires the perspective of people who are working within the system and have a sufficiently nuanced understanding of implementation realities, permitting them to see past the *symptoms* (implementation barriers) and diagnose the underlying *causes* of these symptoms (systems failure). However, it is equally important to secure adequate expertise in scientific inquiry within the teams conducting embedded research in order to ensure that the quality of the research—and hence its credibility and acceptability—is rigorous and sound.

The iPIER experience in the LAC countries, therefore, has highlighted that IR is ideally carried out through a collaborative endeavor in which knowledge is coproduced by implementers and by researchers. The skills and perspectives of both these groups are needed to maximize the impact of IR.

Implementers play an important function by grounding the research in the real world and ensuring that findings are used. By fostering “buy-in” in the development and implementation of program improvements among a broad range of stakeholders, implementers work to ensure the sustainability of the research findings. Implementers are key in defining the main outcomes, facilitating the execution of research, promoting collaboration among different actors, and implementing changes in real time as the problems are identified. For their part, researchers ensure that an appropriate study design and rigorous methods are used to generate robust knowledge for problem-solving.

The involvement of both these sets of actors is also critical in terms of focusing the research. Given the importance of defining a research question that is explicitly centered on issues of *implementation* (4)*,* this can only be achieved if both implementers and researchers contribute their perspectives. By adopting a systems lens, implementers and researchers can together frame the research question to reflect a deliberate consideration of the factors affecting implementation strategies. This embedded research approach is predicated on the direct, active involvement of and collaboration between both these sets of actors, from the outset of the research. Such collaboration helps circumvent many of the known obstacles to research translation (5, 6).

A key advantage of this model arises from the fact that knowledge is coproduced and therefore the intensity of what has traditionally been understood as “dissemination” efforts is reduced. Since the implementers involved in the research have informed its focus and execution, they are also able to insert themselves more easily into the policymaking sphere to extend the reach of the research findings more broadly among other key stakeholders within the health system. The uptake of the evidence produced is greatly facilitated by this fact (7). In some instances, the evidence can be used in real time by the implementers, given their proximity to and influence in the health programs under study.

It is clear from this experience in Latin America and the Caribbean that the nature of the engagement of implementers differs depending on the specific context, level of the health system at which the implementer is working, and the issue being addressed by the research. In some instances, the implementers themselves had backgrounds in research and were able to be much more involved in the conduct of the data collection and analysis. In other instances, they were more heavily engaged in the planning phase and during the interpretation of the results. Regardless of the level and nature of engagement, there was a commitment and willingness among implementers to participate and contribute to the research, despite having other commitments and priorities. By embedding research within the program activities, implementers assumed greater ownership over the research and saw it within their scope of responsibilities.

Lastly, it is important to acknowledge that for this embedded research approach to work, certain minimum capacities are needed within the research team. First, the researchers bring to bear the capacity for strong scientific inquiry. Second, the implementers provide the capacity for leadership/stewardship, dialogue, and engagement of a broad range of stakeholders.

The protocol development and data analysis workshops of the iPIER initiative have focused on building the capacity for strong scientific inquiry. This external support was particularly important in the development of an effective and feasible project, with focused and specific IR objectives rather than the epidemiological lens to which most grantees were accustomed. Furthermore, the research process itself contributes to building the capacity of implementers around research and enhancing collaboration among other stakeholders.

## IMPLICATIONS FOR THE BROADER FIELD OF IMPLEMENTATION RESEARCH

The iPIER experience of embedded research in the LAC countries has provided early indications that this is a promising approach to implementation research. While more formal evaluation efforts to demonstrate the effectiveness of the model are still forthcoming, our experience permits us to put forward a few recommendations in the context of implementation research.

It is clear that strong incentives are needed to motivate and support the collaboration between the implementer and the researcher that we have described. Ideally, these incentives should come from within the health system, in order to promote continuity and institutionalization of this approach without requiring the external funding or technical assistance provided by iPIER. Integrating this practice of coproduction of research into routine health system programming is critical in this regard. This type of integration requires strong evidence of its effectiveness and buy-in among key stakeholders.

Equally important are efforts to strengthen local capacity for this type of embedded research among health system professionals. The aim is not to convert implementers into investigators, or vice versa. However, there is great value in, on the one hand, promoting deeper understanding of research design and methods among implementers and, on the other hand, promoting a more nuanced awareness among researchers about the health system needs and constraints that affect health program practice (8). This embedded research model ultimately requires both parties—researchers and implementers—to adapt their way of doing things and to consider a different perspective. For that to happen, researchers need to be open to practical program considerations, and implementers need to be open to the science of implementation research.

## CONCLUSION

The iPIER experience in Latin America and the Caribbean has shown great promise for embedded research models that place implementers at the helm of IR initiatives. The response to the iPIER call for proposals in Latin America and the Caribbean, with 234 applications submitted, demonstrated that there is a strong interest and willingness to engage in this type of research model by both implementers and researchers. The potential for such an approach to yield concrete benefits in the form of realistic, evidence-informed, broadly accepted health program improvements is great. Given the relatively small size of the awards, this approach could be very attractive to funders, and it should be expanded beyond iPIER.

While rigorous evaluation of the embedded research approach is still forthcoming, the experiences to date demonstrate that it is tractable and informative within the health system decision-making arena. We encourage stakeholders to consider using this embedded research approach to improve their health policies and programs outside the context of iPIER.

### Disclaimer

Authors hold sole responsibility for the views expressed in the manuscript, which may not necessarily reflect the opinion or policy of the *RPSP/PAJPH*, the Alliance for Health Policy and Systems Research, the Pan American Health Organization, or Johns Hopkins University Bloomberg School of Public Health.
